# Clinical Application of Computer-Aided Diagnostic System for Harmonious Introduction of Complementary Dialysis Therapy

**DOI:** 10.2174/1874120700802010010

**Published:** 2008-04-01

**Authors:** Abdullah Al Mamun, Hiroyuki Hamada, Tomokazu Karino, Shinji Namoto, Akihiro C Yamashita, Makoto Ishizaki, Masahiro Okamoto

**Affiliations:** 1 Laboratory for Bioinformatics, Grad. Sch. of Bioresources and Bioenvironmental Sciences, Kyushu University; 2 Laboratory for Bioinformatics, Grad. Sch. of Systems Life Sciences, Kyushu University; 3 Research and Development, JMS Co., Ltd.; 4 Department of Materials Science and Engineering, Shonan Institute of Technology; 5 Eijinkai Hospital, Kidney Center, Japan

**Keywords:** Peritoneal dialysis, Complementary dialysis, Diffusive selectivity index, Computer-aided diagnosis, Therapeutic monitoring.

## Abstract

In chronic peritoneal dialysis (PD) therapy, peritoneal permeability is gradually enhanced over the duration of the therapeutic course, leading to a grave decline in the therapeutic efficiency. In recent years, a novel therapy (CD therapy), which integrates PD therapy with hemodialysis therapy, is being applied to end-stage PD patients to complement the decline of therapeutic efficiency caused by the grave degeneration of the peritoneal tissue. To realize a harmonious introduction of the CD therapy, this study developed a useful index (*KAu/c*), which evaluates both therapeutic efficiency and degeneration of peritoneal tissue. Using a mathematical model and *KAu/c*, we were able to validate the therapeutic efficiency in the PD patients, and, in one case, propose a better prescription for the patient by employing the CD therapy. The clinical implementation of this methodology is indispensable with regard to expanding the therapeutic monitoring system for renal replacement therapy.

## INTRODUCTION 

Chronic peritoneal dialysis (PD) therapy is a well-known renal replacement therapy and has been widely used in the world in conjunction with chronic hemodialysis (HD) therapy, as its clinical implementation provides support in many end-stage renal disease patients [1]. In chronic PD therapy, a slightly hyperosmotic washing solution called dialysate, is put into the peritoneal cavity of a patient using a peritoneal catheter, which is implanted into the abdominal cavity. The dialysate is exchanged four to five times a day by the patient. By employing the principles of osmotic flow, diffusive and convective transport, the metabolites and excess water in the body fluids are gradually removed to the dialysate. Compared to patients on chronic HD therapy, residual renal function is better preserved in patients who are on chronic PD therapy [2,3]. Moreover, the dialysis outcome for the middle molecules is also superior for chronic PD therapy as compared to that seen for chronic HD therapy [2,3].

However, the peritoneal permeability gradually enhances with the course of therapeutic duration, which leads to a grave decline in both dialysis outcomes and excess water removal volume (therapeutic efficiency). Hence, it is extremely important to explore a better prescription for chronic PD therapy based on both the peritoneal permeability and the residual renal function. However, in most cases, patients are actually being treated without ever having any rigorous therapeutic monitoring of the peritoneal permeability.

In recent years, a novel therapy (complementary dialysis therapy: CD therapy) that integrates PD therapy with HD therapy has been applied to end-stage PD patients as a way of preventing the decline of the therapeutic efficiency related to the grave degeneration of the peritoneal tissue [4]. In the CD therapy, the weekly regimen of patient is scheduled for one day of HD therapy after 5 or 6 days of PD therapy. Since the CD therapy combines PD therapy’s maintenance of body fluid homeostasis with the excellent therapeutic efficiency of the HD therapy, end-stage PD patients who show a decline in therapeutic efficiency caused by the grave degeneration of the peritoneal tissue exhibit improvements in both therapeutic efficiency and quality of life when started on the CD therapy [5]. In addition, the CD therapy is particularly available for a transition between full time PD therapy and full time HD therapy, and makes it possible to alleviate the degradation of peritoneal tissue by an effect of peritoneal rest produced by intermittent HD therapy [6,7]. The introduction of CD therapy is based on the dialysis outcomes of PD therapy such as creatinine clearance (*CCr*) and *Kt/V* for urea. However, during PD therapy, artificial errors in clinical data such as drainage volume and residual abdominal dialysate volume can occur when patients exchange dialysate, which can greatly affect the reproducibility of the dialysis outcomes. Furthermore, the multifarious therapeutic conditions that are available for PD therapy can also have an extreme influence on the dialysis outcomes, thus making it difficult to evaluate the degeneration of the peritoneal tissue by only using the dialysis outcomes. Therefore, development of a useful index that is capable of evaluating both dialysis outcomes and the degeneration of the peritoneal tissue would be indispensable in being able to realize a harmonious introduction of CD therapy.

A numerical analysis that employs a mathematical model for chronic PD therapy is one of the quantitative assessments for peritoneal permeability, and is useful for developing the indexes. Assuming that the peritoneum was homoporous, Popovich *et al*. constructed a mathematical model that were able to quantitatively estimate the mass transfers of both crystalloids and colloids during chronic PD therapy (Pyle-Popovich’s model) [8]. In order to accurately represent the osmotic flow, Rippe *et al*. constructed a mathematical model based on a heteroporus theory (three-pore theory) [9]. Vonesh *et al*. applied the concept of convective transport from the three-pore theory to the convective term of Pyle-Popovich’s peritoneal dialysis model, thereby making it possible to estimate the dialysis outcomes for each of the patients (Vonesh’s model) [10]. By integrating Pyle-Popovich’s model with the three-pore theory as well as incorporating Vonesh’s model, Hamada *et al*. were able to construct a mathematical model for chronic PD therapy that led to the development of intelligent software, which resulted in better prescriptions for chronic PD therapy (Peritoneal Dialysis Navigation: PD NAVI^®^) [11-16]. In addition, the new software implemented a numerical simulator that can be used to estimate both the dialysis outcome and excess water removal volume of the CD therapy (Peritoneal and Hemo Dialysis Navigation: PHD NAVI^®^) [17]. By using a simultaneous numerical optimization technique, these tools are able to determine a set of optimal kinetic parameters for each patient’s clinical condition, thereby supporting the realization of an individualized therapy. Some of the parameters are defined based on the morphological findings of the peritoneal tissue and are available for use in evaluating the peritoneal permeability. Therefore, products that employ these parameters are useful in the therapeutic monitoring of both the decline of dialysis outcomes and the enhancement of peritoneal permeability caused by the degeneration of peritoneal tissue, and have prospects of a useful index that assess an introduction of the CD treatment.

In the current paper, we propose a novel index employing kinetic parameters that evaluate both the dialysis outcomes and the peritoneal permeability, which allows for the verification of an optimal introduction of the CD therapy. Then, by using the novel index, PD NAVI^®^ and PHD NAVI^®^ are integrated in order to increase the availability of a computer-aided diagnostic system for harmonious introduction of complementary dialysis therapy. Moreover, the proposed system analyzes some clinical data for which the peritoneal permeability differs from patient to patient, and gives a better prescription to a clinical case for which the novel index indicated an alert state. The clinical implementation of this methodology is an indispensable way to realize a suitable introduction of CD therapy and to develop a therapeutic monitoring system for chronic PD therapy.

## MATERIALS AND METHODS

### 1. Kinetics for Chronic PD Therapy

A mathematical model that is capable of performing numerical simulations of chronic PD therapy is now available for making diagnostic assessments such as the monitoring of peritoneal permeability and verifications of prescriptions [13-15]. In the current study, we developed a novel index that was able to evaluate both dialysis outcomes and the peritoneal permeability involving the degeneration of the peritoneal tissue. To accurately evaluate membrane transport, this study adopted a precise model [16] for which the concept of convective transport from the three-pore theory [9] was applied to the convective term of Pyle-Popovich’s model [8]. The three-pore theory considers three kinds of pores, each with a different radius size on the capillary wall; *i.e.*, *Cell pore*, *Small pore* and *Large pore*. *Cell pore* corresponds to the water channel (Aquaporin). When employing both diffusive and convective transport, crystalloids and colloids are removed to the dialysate via the *Small pore* or *Large pore*, respectively. The equations for the models are as follows.

… (1) $$\frac{{\mathrm{dV}}_{B}C_{\mathrm{BX}}}{\mathrm{dt}}=G_{X}-\frac{{\mathrm{dV}}_{D}C_{\mathrm{DX}}}{\mathrm{dt}}-C_{\mathrm{LrX}}.C_{\mathrm{BX}}$$

… (2) $$\frac{{\mathrm{dV}}_{D}C_{\mathrm{DX}}}{\mathrm{dt}}=\dot{m_{X}}={\mathrm{KA}}_{X}.(C_{\mathrm{BX}}-C_{\mathrm{DX}})+Q_{\mathrm{UP}}.(1-\sigma_{X}).{\overset{-}{C}}_{X}$$

where the metabolite *X* of interest is denoted by the subscript *X, *corresponding to urea, creatinine, glucose, albumin and so forth. *V_B_* and *V_D_* are the total body fluid volume and intraperitoneal dialysate volume (mL), respectively. *V_B_* implies the space including the metabolite *X* of interest. *C_B_* and *C_D_* are the well-mixed plasma and dialysate concentration (mg/mL), respectively. *C_Lr_* is the residual renal clearance (mL/min) and *G* is the generation rate (mg/min). $\overset{\cdot }{m}$ is the mass transfer rate for the peritoneal membrane (mg/min). *KA* is known as the overall mass transfer area coefficient (MTAC) (mL/min), *σ* is the reflection coefficient (-), which can quantitatively evaluate the efficiency of the sieve for the convection transport, and $\overset{-}{C}$ is the mean metabolite concentration found within the peritoneal membrane (mg/mL). *Q_UP_* is the osmotic flow rate of the *Small* or *Large pores* for transport of metabolite *X* of interest (mL/min). The net ultrafiltration volume and *Q_UP_* were estimated by employing the three-pore theory, which was defined by the following equations.

… (3) $$Q_{U}=Q_{\mathrm{UC}}+Q_{\mathrm{US}}+Q_{\mathrm{UL}}-\mathrm{Lymph}$$

… (4) $$Q_{\mathrm{UC}}=L_{P}S_{C}.\left(\mathrm{HP}+\pi_{\mathrm{onc}}+\pi_{\mathrm{Glu}}+\pi_{\mathrm{Urea}}+\pi_{\mathrm{Crea}}+\pi_{\mathrm{Na}}+\pi_{\mathrm{Cl}}\right)$$

… (5) $$Q_{\mathrm{US}}=L_{P}S_{S}\;(\mathrm{HP}+\pi_{\mathrm{onc}}+{\sigma \pi }_{S,\mathrm{Cry}}+{\sigma \pi }_{S,\mathrm{Ele}})$$

… (6) $${\sigma \pi }_{S,\mathrm{Cry}}=\sigma_{S,\mathrm{Glu}}\pi_{\mathrm{Glu}}+\sigma_{S,\mathrm{Urea}}\pi_{\mathrm{Urea}}+\sigma_{S,\mathrm{Crea}}\pi_{\mathrm{Crea}}$$

… (7) $${\sigma \pi }_{S,\mathrm{Ele}}=\sigma_{S,\mathrm{Na}}\pi_{\mathrm{Na}}+\sigma_{S,\mathrm{Cl}}\pi_{\mathrm{Cl}}$$

… (8) $$Q_{\mathrm{UL}}=L_{P}S_{L}\left(\mathrm{HP}+\sigma_{L,\mathrm{Onc}}\pi_{\mathrm{Onc}}+{\sigma \pi }_{L,\mathrm{Cry}}+{\sigma \pi }_{L,\mathrm{Ele}}\right)$$

… (9) $${\sigma \pi }_{L,\mathrm{Cry}}=\sigma_{L,\mathrm{Glu}}\pi_{\mathrm{Glu}}+\sigma_{L,\mathrm{Urea}}\pi_{\mathrm{Urea}}+\sigma_{L,\mathrm{Crea}}\pi_{\mathrm{Crea}}$$

… (10) $${\sigma \pi }_{L,\mathrm{Ele}}=\sigma_{L,\mathrm{Na}}\pi_{\mathrm{Na}}+\sigma_{L,\mathrm{Cl}}\pi_{\mathrm{Cl}}$$

… (11) $$L_{P}S=L_{P}S_{C}+L_{P}S_{S}+L_{P}S_{L}$$

… (12) $$V_{D}=V_{D}(0)+\int_{0}^{t}Q_{U}\mathrm{dt}$$

… (13) $$V_{B}=V_{B}(0)+V_{D}(0)-V_{D}-V_{U}$$

where *Q_U_* is the net ultrafiltration rate (mL/min), and *Q_UC_*, *Q_US_* and *Q_UL_* are the osmotic flow rates (mL/min) for the *Cell pore*, *Small pore* and *Large pore*, respectively. *Lymph* is the lymphatic absorption rate (mL/min). *L_P_S_C_*, *L_P_S_S_* and *L_P_S_L_* are the hydraulic conductance (mL/min/mmHg) for the *Cell pore*, *Small pore* and *Large pore*, respectively, and *HP* is the hydrostatic pressure (mmHg). *σ_S_* and *σ_L_* are the reflection coefficient (-) for the *Small pore* and *Large pore*, respectively. Colloid osmotic pressure (mmHg) is represented by *π_One_*. The crystalloid osmotic pressures (mmHg) are *π_Urea_*, *π_Crea_*, etc., and are calculated using van’t Hoff’s equation. *V_U_* is the urine volume (mL). *G*, *KA*, *L_P_S_C_*, *L_P_S_S_*, *L_P_S_L_*, *Lymph* and *HP* are a set of unknown kinetic parameters, and need to be determined from the clinical data for each patient through the use of a simultaneous numerical optimization method [16]. This study numerically calculated a mathematical model, and estimated the time courses for both the concentration of metabolites and the intraperitoneal dialysate volume [11-16].

### 2. Diffusive Selectivity Index

Some of the kinetic parameters for the chronic PD therapy mathematical models are defined based on the features of the peritoneal tissue. Multiplying the overall mass transfer coefficient ( *K* ) by the peritoneal effective area (*A*) gives MTAC, which makes it possible to evaluate the peritoneal permeability based on the diffusive transport. For the chronic PD therapy, small solute removal is extremely dependent upon the diffusive transport in contrast to that required for the convective transport. Since peritoneal tissue degeneration affects the membrane transport, therapeutic monitoring of *K* for small solute makes it possible to observe the enhancement of the peritoneal permeability caused by the degeneration of the peritoneal tissue. Assuming the plasma concentrations of the metabolites in the chronic PD patients are constant within a single dialysate exchange, an approximated analytical solution for MTAC can be calculated based on equation (2) and as shown in following algebraic expression.

… (14) $${\mathrm{KA}}_{X}=-\frac{V_{D}(t)}{t}\text{ln}\left|{\left\{\frac{V_{D}(t)}{V_{D}(0)}\right\}}^{n}\frac{C_{\mathrm{DX}}(t)-C_{\mathrm{BX}}}{C_{\mathrm{DX}}(0)-C_{\mathrm{BX}}}\right|$$

where *t* is the dwelling time (min), *V_D_*(0) is the infusion volume, and *C_DX_*(0) is the dialysate concentration of metabolite immediately after infusion. *n* implies a contribution of convective transport compared to diffusive transport, and is set equal to either 0.0, 0.5 or 1.0 [18-21]. In this study, *n* was set equal to 0.0 due to analyze the mass transfers of small solute such as urea and creatinine. Then, the approximated dialysis outcome for the small solute was calculated by employing equation (15), which was based on equation (14) [22].

… (15) $$\frac{V_{D}(t)C_{\mathrm{DX}}(t)}{C_{\mathrm{BX}}}=V_{D}(t)\left\{1-\text{exp}\left(-\frac{{\mathrm{KA}}_{X}}{V_{D}(t)}t\right)\right\}$$

where the left hand side of equation (15) corresponds to the clearance that evaluates the adequacy of the dialysis during the renal replacement therapy. Although equation (15) theoretically showed a relationship between the clearance and MTAC, it did not prove to be useful clinically due to the lack of normalization. As a result, this study defined the ratio (*KAu/c*) of the *KA* for urea to *KA* for creatinine, and assessed the relationship between *KAu/c* and therapeutic efficiency.

… (16) $$\mathrm{KAu}/c=\frac{{\mathrm{KA}}_{\mathrm{urea}}}{{\mathrm{KA}}_{\mathrm{creatinine}}}=\frac{\text{ln}\left\{1-\frac{\mathrm{Kt}}{V_{D}(t)}\right\}}{\text{ln}\left\{1-\frac{\mathrm{CCr}}{V_{D}(t)}\right\}}$$

where *Kt* and *CCr* are urea clearance and creatinine clearance, respectively. *KAu/c* implies a peritoneal diffusive selectivity (Diffusive Selective Index), and is a dimensionless parameter which accurately evaluates a personal equation for the clinical data. The enhancement of peritoneal permeability based on the degeneration of peritoneal tissue increases the overall mass transfer coefficient (*K*) for both urea and creatinine and decreases a ratio of *K* for urea to that for creatinine. Therefore, *KAu/c* for High transporter is normally much larger than that for other transporters. As for the clearances, the CANUSA study and the NKF-K/DOQI guidelines proposed clinical practice recommendations, which imply minimum requirements for the adequacy of dialysis on the chronic PD therapy [23-26]. The relationship between *KAu/c* and therapeutic efficiency was validated by applying the clinical practice recommendations to equation (16).

### 3. Kinetics for HD Therapy

The Urea Kinetic Model [27] was applied to the HD therapy. The set of mass balance equations for the plasma concentration on the HD therapy was as follows.

… (17) $$\frac{{\mathrm{dV}}_{I}C_{\mathrm{IX}}}{\mathrm{dt}}=G_{\mathrm{IX}}-\frac{{\mathrm{dV}}_{E}C_{\mathrm{EX}}}{\mathrm{dt}}=G_{\mathrm{IX}}-K_{\mathrm{CX}}\left(C_{\mathrm{IX}}-C_{\mathrm{EX}}\right)$$

… (18) $$\frac{{\mathrm{dV}}_{E}C_{\mathrm{EX}}}{\mathrm{dt}}=G_{\mathrm{EX}}+K_{\mathrm{CX}}\left(C_{\mathrm{IX}}-C_{\mathrm{EX}}\right)-C_{\mathrm{LDX}}C_{\mathrm{EX}}-C_{\mathrm{LrX}}C_{\mathrm{EX}}$$

where *V_I_* and *V_E_* are the intracellular fluid volume (mL) and the extracellular fluid volume (mL). *C_I_* and *C_E_* are the intracellular concentration (mg/mL) and the extracellular concentration (mg/mL). *G_I_* and *G_E_* are the intracellular generation rate (mg/min) and the extracellular generation rate (mg/min). *K_C_* and *C_LD_* are the clearance of the cellular membrane (mL/min) and clearance of the dialyzer membrane (mL/min). *V_I_*, *V_E_*, *G_I_*, *G_E_*, *K_C_* and *C_LD_* for each patient were determined by using empirical equations [17]. This study numerically calculated a mathematical model, and estimated the time courses for the concentration of the metabolites [17]. Since *C_LD_* is rather larger than *K_C_*, the time course for *C_I_* showed a considerable discrepancy versus that seen for *C_E_*. Hence the total body fluid for the HD therapy was divided into two compartments: i.e., intracellular fluid and extracellular fluid.

### 4. Kinetics for CD Therapy

The numerical simulations for the CD therapy were executed by employing a set of mathematical models for both chronic PD and HD therapies. The dialysis outcomes for the CD therapy were estimated by employing weekly Clear Space (*CS*), which allows for the integration of the dialysis outcome for chronic PD therapy with that of HD therapy [17]. The *CS*, which divided quantity of solute removal by metabolite concentration in plasma, corresponds to the purified body fluid volume during the CD therapy.

However *CS* was not normalized as a universal therapeutic index, so that this study evaluated the dialysis outcome by employing the *CS*/*V_B_*, which cancels out individual differences between patients. The *CS*/*V_B_* was a dimensionless parameter, and may set the criterion that is common to all patients. Since the *CS* was quantitatively similar to the *CCr* implemented for PD, *CS* and *CS*/*V_B_* were evaluated based on the clinical practice recommendations for *CCr* and *Kt*/*V* for Urea on the chronic PD therapy [23-26]. The total amount of the fluid removal summed the weekly urine volume and the total ultrafiltration volume of CD therapy in a weekly regimen. In addition, the time course of the metabolite concentrations in plasma after having prescribed an arbitrary treatment schedule was estimated by employing a set of mathematical model for both the chronic PD and HD therapy.

### 5. Peritoneal Function Test

In order to validate our methodology, three Japanese patients undergoing the chronic PD therapy underwent the peritoneal function tests shown in Fig. (**1**), leading to the accumulation of sets of essential clinical data. There were multiple dialysate samples collected, and these included two kinds of dialysate for which there were differing osmotic pressures i.e., a 360-mOsm/kg-solvent (Low-Sol’n) and a 400-mOsm/kg-solvent (Mid-Sol’n). The essential clinical data collected for use in the unknown parameters optimization are shown in Table **1**. The clinical tests that involved the PET [28] were scheduled so that there were three exchanges of Low-Sol’n and Mid-Sol’n, for which the dwelling times differed. The patient’s backgrounds are seen in Table **2**. Since a management of total body fluid volume on CD therapy depends on the chronic PD therapy, body surface area and total body fluid volume were estimated by employing the Gehan equation [29], and the Hume and Weyers equation [30], respectively. These equations are available for kinetics for the chronic PD therapy [24-26]. All patients signed informed consents that allowed for the use of their clinical data in the studies that were conducted to validate the clinical effectiveness of the proposed methodology.

## RESULTS AND DISCUSSION

The accuracy of the mathematical model that were used to run a numerical simulation for chronic PD therapy was verified by using the clinical data acquired based on the peritoneal function test shown in Fig. (**1**). A simultaneous numerical optimization technique for the PD NAVI^®^ determined a set of unknown kinetic parameters. The mass transfer for the peritoneum was numerically estimated by applying the optimal kinetic parameters to a mathematical model for the chronic PD therapy. Fig. (**2**) showed a relationship between measured and calculated values of *Kt/V* for urea, *CCr* and drainage volume. The coefficients of determination for both *Kt/V* for urea and *CCr* were very high (R^2^>0.92), which showed that calculated data were in good agreement with measured data. Therefore, the simultaneous numerical optimization technique on PD NAVI^®^ accurately determined a set of kinetic parameters related to mass transfer, and guaranteed an availability of analysis of the peritoneal permeability employing *KA* for both urea and creatinine. On the other hand, the coefficient of determination for drainage volume (R^2^>0.86) was lower than those for both *Kt/V* for urea and *CCr*. When patients exchange dialysate, clinical data such as drainage volume and residual abdominal dialysate volume can easily include unavoidable artificial errors involving a daily variance. Consequently, the calculated data of drainage volume showed a slight discrepancy with the measured data, which had an effect on the coefficient of determination for the drainage volume. As a result, the calculated data of drainage volume were in good agreement with the measured data, and a set of kinetic parameters related to hydraulic conductance was also determined by employing the simultaneous numerical optimization technique on PD NAVI^®^. Thus, as calculated by the PD NAVI^®^, there was little discrepancy for both dialysis outcome and fluid removal volume as compared to the measured data shown in Fig. (**2**). Since the numerical simulation accurately estimated the dialysis outcome and fluid removal volume for the chronic PD therapy, the numerical analysis by PD NAVI^®^ can be used to validate the relationship between *KAu/c* and the therapeutic efficiency such as *Kt/V* for urea, *CCr* and drainage volume.

The dialysis outcome and fluid removal volume for each patient based on the prescriptions shown in Fig. (**1**) were compared with the clinical practice recommendations [24-26] in order to determine the adequacy of the dialysis (Table **3**). Although the dialysis outcomes and fluid removal volume for both Patients 1 and 2 were sufficient to earn an adequacy of dialysis designation, those for Patient 3 were equal to the clinical practice recommendations, which implies that a better prescription needs to be explored for this patient. Moreover, therapeutic efficiency for each patient was compared using the* KAu/c*, which was determined by applying each patient’s clinical data to the PD NAVI^®^ (Table **3**). With decreases of *KAu/c* there was an associated decrease in the dialysis outcomes, which qualitatively was in good agreement with the peritoneal permeability properties that were based on the PET. *KAu/c* is a ratio of the *KA* for urea to the *KA* for creatinine, and is a dimensionless parameter that does not involve the effective peritoneal area. Since the enhancement of the peritoneal permeability that is caused by the degeneration of the peritoneal tissue increases the overall mass transfer coefficient (*K*) and decreases the* KAu/c*, therapeutic monitoring of *KAu/c* makes it possible to observe both the enhancement of the peritoneal permeability and the degeneration of the peritoneal tissue. In addition, the *KAu/c* has the potential for use in meta-analyses on chronic PD therapy administered in multinational and multicenter studies, for which the clinical conditions differ from patient to patient.

The theoretical approach for chronic PD therapy that is based on an approximated analytical solution of a mathematical model is useful for the elucidation of some of the properties of *KAu/c*. When *KAu/c *is defined as an independent variable in equation (16), one can numerically analyze a relationship between *KAu/c* and the therapeutic efficiencies. As seen in Fig. (**3**), the improvement of the dialysis outcomes depended on increases of both *KAu/c* and the drainage volume. Moreover, the theoretical relationship is available for verification of a feasibility of adequacy of dialysis based on each patient clinical data. In Fig. (**3**), both drainage volume and *KAu/c* for each patient in Table **3** were plotted on the relationship. Although both Patient 1 (low transporter) and Patient 2 (average transporter) achieved an adequacy for which the dialysis outcomes fully exceeded the minimum clinical practice recommendation (solid line), results for Patient 3 (high transporter) corresponded to the minimum clinical practice recommendations, implying that a better prescription for the patient needed to be pursued. These findings were in agreement with the results presented in Table **3**. However, as noted in Fig. (**3**), in order to increase the *Kt/V* by 0.15, it would be necessary to increase the drainage volume by over 2.0 L per day in Patient 3. In addition, since the peritoneal permeability gradually enhances during the course of the therapeutic duration, maintaining high transporters such as Patient 3 on chronic PD therapy would be difficult at best to achieve. Thus, unfortunately, Fig. (**3**) implies that it is extremely difficult to determine better prescriptions for high transporters as compared to the other transporters. By employing a relationship shown in Fig. (**3**) for the purpose of the therapeutic monitoring of* KAu/c*, this makes it possible to easily evaluate the adequacy of the dialysis and in addition, provides useful findings on the therapeutic strategies. As for cases similar to the patient examined in this study, CD therapy plays an important role in improving the dialysis outcome and fluid removal volume.

The therapeutic efficiency for the CD therapy for Patient 3 in Table **3** was estimated by applying the clinical data to the PHD NAVI^®^. The treatment schedule for the PD prescription for the CD therapy is shown in Fig. (**1**). Table **4** shows the therapeutic condition for HD during the CD therapy. In Table **5**, the relationship between the weekly regimen and the therapeutic efficiency can be seen, with R1 scheduled for 7 days of PD, R2 scheduled for one day of HD and then a dialysis holiday after 5 days of PD, and R3 scheduled for one day of HD after 6 days of PD. In addition, the dialysis outcomes were estimated by using *CS* [17]. Although dialysis outcomes for R2 increased more than those for R1, the fluid removal volume was below the clinical practice recommendations. However, the dialysis outcome and fluid removal volume for R3 completely met the clinical practice recommendations, which proved to be an effectual regimen for Patient 3. Thus, the CD therapy complements the decline of the therapeutic efficiency observed during chronic PD therapy by effectively employing the HD therapy, which is very useful in the realization of a long-term PD therapy. Moreover, numerical analyses that apply the PHD NAVI^®^ make it possible to easily explore better prescriptions and regimens for CD therapy with minimal nursing time. The monitoring of *KAu/c* by employing the relationship shown in Fig. (**3**) is indispensable for being able to realize a harmonious introduction of the CD therapy. The computer-aided diagnosis, which integrates the PD NAVI^®^ with *KAu/c* and PHD NAVI^®^, is available for both the verification of adequacy and the validation of the regimen for the chronic PD therapy that involves the CD therapy. When taken together, these may play an important role in the improvement of the quality of life for chronic PD patients.

## CONCLUSIONS

A novel criterion (*KAu/c*), which evaluates the peritoneal permeability caused by the degeneration of the peritoneal tissue, was proposed by using a mathematical model for chronic PD therapy. Analysis of some of the clinical data, for which patient to patient peritoneal permeability differed, was performed in order to validate the clinical usefulness of *KAu/c*. The dialysis outcome and fluid removal volume decreased with the decrease of* KAu/c*, which qualitatively was in good agreement with the properties of the peritoneal permeability that was based on PET. Moreover, the therapeutic monitoring of *KAu/c* made it possible to easily evaluate the adequacy of the dialysis and provided useful findings on potential therapeutic strategies. For one critical case, in which it proved difficult to keep the patient on the chronic PD therapy, we explored the possibilities for a better CD therapy prescription, and by using this method, we numerically demonstrated that the CD therapy complemented the decline of both the dialysis outcome and fluid removal volume that occurred for the chronic PD therapy by effectively employing the HD therapy. The computer-aided diagnosis which integrates PD NAVI^®^ with *KAu/c* and PHD NAVI^®^ is available for both verifying the adequacy and the validation of the regimen of chronic PD therapy that involves CD therapy, and in the future may contribute to continued expansion of renal replacement therapies.

## Figures and Tables

**Fig. (1):**
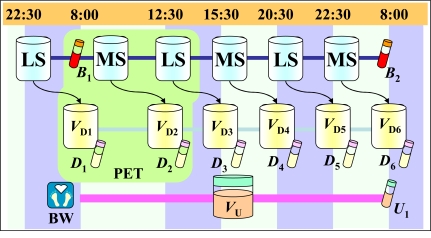
Timetable of the peritoneal function test. LS: low osmotic pressure solution; MS: middle osmotic pressure solution; BW: body weight; B_1_-B_2_: blood samples; VD_1_~ VD_6_: drain-age; D_1_~ D_6_: drainage samples; V_U_: urine; U_1_: urine sample; PET: peritoneal equilibrium test [28]. Infusion volume of the dialysate is 2.0 L.

**Fig. (2):**
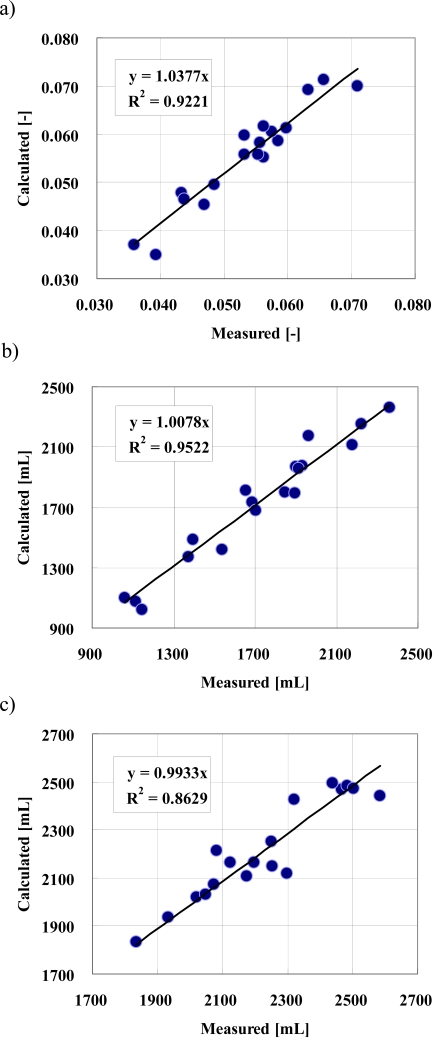
Measured (actual) versus calculated values of dialysis outcome and drainage volume. These data correspond to the therapeutic efficiencies for a single exchange of dialysate. a) *Kt/V* for urea, b) *CCr*, c) drainage volume. The coefficients of determination for both *Kt/V* for urea and *CCr* were very high (R^2^>0.92). Meanwhile, the coefficient of determination for drainage volume (R^2^>0.86) was lower than others. Since drainage volume can easily include unavoidable artificial errors, the calculated data showed a slight discrepancy with the measured data of drainage volume. As a result, the calculated data showed excellent agreement with the measured data, and a set of kinetic parameters was exactly determined by employing the simultaneous numerical optimization technique.

**Fig. (3):**
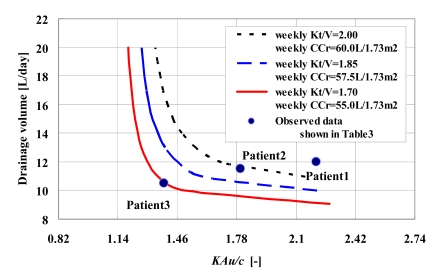
Verification of adequacy of dialysis using a theoretical relationship between drainage volume and *KAu/c*. When there were increases for both the drainage volume and *KAu/c*, dialysis outcome and fluid removal volume improved. Although both Patient 1 and Patient 2 achieved an adequacy of dialysis, Patient 3 corresponded to the minimum clinical practice recommendations [24-26]. Solid line is the curve for the minimum clinical practice recommendations. Patient 1, Patient 2 and Patient 3 correspond to the clinical data shown in Table **3**.

**Table 1:** Measurement Data

Dwell time	Inf. vol.	UF vol.
Solute concentrations (Plasma, Dialysate and Urine)		
urea	creatinine	glucose
albumin	Na	Cl

Dwell time (min). Inf. vol.: Infusion volume (L). UF vol.: Ultrafiltration volume (L).

**Table 2:** Patients

Age	50.3±6.4
Male:Female	2 : 1
BSA	1.71±0.16 m^2^
TBFV	36.0±6.5 L
Urine	78.3±109.8 mL/day

BSA: Body Surface Area [29]. TBFV: Total Body Fluid Volume [30].

**Table 3:** Relationship Between Adequacy of Dialysis and *KAu/c*

		Pt1	Pt2	Pt3	CPR
*Kt/V*	[-]	2.15	1.78	1.73	>1.70
*CCr*	[L/1.73m^2^]	61.6	60.4	54.2	>55.0
FRV	[L]	14.0	10.5	3.5	> 4.9
*KAu/c*	[-]	2.21	1.80	1.39	
PET		Low	Ave	High	

*Kt/V*: Weekly *Kt/V* for urea. *CCr*: Weekly creatinine clearance. FRV: Weekly fluid removal volume. CPR: Clinical practice recommendations [24-26].

**Table 4:** Therapeutic Conditions on HD Therapy

Treatment time	240min
Ultrafiltration rate	8.33mL/min
Urea clearance	170mL/min
Creatinine clearance	150mL/min

**Table 5:** Regimens and Therapeutic Efficiency on CD Therapy

		R1	R2	R3	CPR
PD	[day]	7	5	6	
HD	[day]	0	1	1	
DH	[day]	0	1	0	
*CSu/V_B_*	[-]	1.73	2.18	2.46	>1.70
*CSc*	[L/1.73m^2^]	54.2	75.2	86.3	>55.0
FRV	[L]	3.5	4.5	5.0	>4.9

PD: Chronic peritoneal dialysis therapy. HD: Intermittent hemodialysis therapy. DH: Dialysis holiday. FRV: Weekly fluid removal volume. *CSu/V_B_*: Ratio of weekly CS for urea to total body fluid volume. *CSc*: Weekly CS for creatinine. CPR: Clinical practice recommendations [24-26].
